# Association of knee and hip osteoarthritis with the risk of falls and fractures: a systematic review and meta-analysis

**DOI:** 10.1186/s13075-023-03179-4

**Published:** 2023-09-29

**Authors:** Youyou Zhang, Xiaoxi Li, Yining Wang, Liru Ge, Faming Pan, Tania Winzenberg, Guoqi Cai

**Affiliations:** 1 Department of Epidemiology and Biostatistics, School of Public Health, Anhui Medical University, Hefei, 230032 Anhui China; 2 Inflammation and Immune Mediated Diseases Laboratory of Anhui Province, Hefei, 230032 Anhui China; 3 School of Medicine, University of Tasmania, Hobart, TAS Australia; 4 Menzies Institute for Medical Research, University of Tasmania, Hobart, TAS Australia

**Keywords:** Falls, Fractures, Hip osteoarthritis, Knee osteoarthritis, Recurrent falls

## Abstract

**Objective:**

Studies evaluating the association of knee and hip osteoarthritis (OA) with falls and fractures have inconsistent findings. We aimed to investigate associations of symptomatic and radiographic knee and hip OA with risk of falls, recurrent falls, and fractures.

**Methods:**

We conducted an electronic search of databases from inception to February 2023. Two authors independently screened studies, extracted data, and assessed the risk of bias using the Newcastle-Ottawa Scale tool in eligible studies. Pooled odds ratios (ORs) with 95% confidence intervals (CIs) were calculated using random-effects models.

**Results:**

Of 17 studies included (*n* = 862849), 2 had a high risk of bias. Among studies that evaluated falls or fractures as outcomes, 7/8 (87.5%) and 5/11 (45.5%) were self-reported, respectively. Both symptomatic knee and hip OA were associated with increased risk of recurrent falls (knee: OR = 1.55, 95% CI 1.10 to 2.18; hip: OR = 1.50, 95% CI 1.28 to 1.75) but not falls or fractures. Radiographic knee OA increased risk of falls (OR = 1.28, 95% CI 1.03 to 1.59) and did not significantly increase risk of recurrent falls (OR = 1.39, 95% CI 0.97 to 1.97) or fractures (OR = 1.22, 95% CI 0.99 to 1.52). Radiographic hip OA decreased the risk of recurrent falls (OR = 0.70, 95% CI 0.51 to 0.96) but had no statistically significant association with fractures (OR = 1.16, 95% CI 0.79 to 1.71).

**Conclusion:**

Symptomatic knee and hip OA were both associated with an increased risk of recurrent falls, and radiographic knee OA was associated with an increased risk of falls. No statistically significant associations of radiographic and symptomatic knee or hip OA with fractures were found.

**Supplementary Information:**

The online version contains supplementary material available at 10.1186/s13075-023-03179-4.

## Key messages

Both symptomatic knee and hip OA were associated with an increased risk of recurrent falls.

Radiographic knee OA was associated with an increased risk of falls.

Symptomatic OA should be considered as potential risk factors for falls and falls risk assessment.

**Supplementary Information:**

The online version contains supplementary material available at 10.1186/s13075-023-03179-4.

## Introduction

Falls and osteoarthritis (OA) are both major public health problems. Falls are the second leading cause of unintentional injury deaths worldwide, and 37.3 million falls requires medical attention each year [1]. As a major consequence of falls, fractures lead to significant mortality and morbidity and socioeconomic burden [2]. OA is the most common joint disorder characterized by joint symptoms (e.g. pain and functional disability) and joint structural changes. The incidence of falls and fractures increases with age [3], and knee OA and hip OA are also highly prevalent in older population [4]. Nearly 30% of individuals older than 45 years have radiographic evidence of knee OA, and about half have knee symptoms [5, 6]. Patients with knee and hip OA frequently have pain, muscle weakness, impaired joint proprioception, and poor balance [7, 8], which are important risk factors for falls.

Studies evaluating the relationship of knee OA and hip OA with falls and fractures have inconsistent findings. Some reported that radiographic or symptomatic OA of the knee and the hip increased the risk of falls, recurrent falls, or fractures [9–15], one suggested that symptomatic knee OA decreased the risk of fractures [16]. For hip OA, one study found that women with radiographic hip OA reduced the risk of recurrent falls [17], others showed that radiographic and symptomatic hip OA were associated with a reduced risk of fractures [18, 19]. Therefore, systematic review and evaluation of quality of evidence is need for the association between OA and the risk of falls and fractures.

Though there is a recent meta-analysis (search date March 2020) examining the relationship of radiographic and self-reported OA with the risk of falls [20], there is no such review for fracture outcomes. The falls review also had methodological limitations as it pooled results of knee and hip OA and of radiographic and symptomatic (self-reported) OA together, which may have introduced biases [20] given that significant differences between knee and hip OA have been identified in many aspects [21]. For example, the experience of pain is different between hip OA and knee OA [21], instability in hip OA may be more likely to occur than instability in knee OA [22]. Moreover, there is discordance between radiographic and symptomatic OA [23, 24], and unadjusted data were combined with results from adjusted models, which may have introduced confounding. Also, new studies evaluating the associations of OA with falls and fractures have been published since this meta-analysis [10, 11, 25]. Therefore, this study aimed to separately determine the associations of symptomatic and radiographic knee OA and hip OA with falls, recurrent falls, and fractures.

## Methods

### Protocol registration

The protocol for the systematic review and meta-analysis was registered with PROSPERO (https://www.crd.york.ac.uk/PROSPERO/, CRD42022311465). It is reported according to the Preferred Reporting Items for Systematic Reviews and Meta-Analyses checklist [26].

### Search strategy

We searched MEDLINE (via Ovid), EMBASE (via Ovid), and Web of Science databases from inception to October 2021 and updated tour search in February 2023, for relevant studies focusing on the association of knee and hip osteoarthritis in the risk of falls and fractures. The search strategies are provided in the Supplementary Methods. We also checked the bibliographies of the original studies and relevant systematic reviews and gray literature (e.g. conference abstracts) for additional studies.

### Study selection

Two authors (YZ and XL) independently reviewed the titles and abstracts of all identified studies and retrieved full texts of relevant studies for further screening. Full-text reviews were conducted following the a priori selection criteria detailed in the registered protocol. Inclusion criteria included: 1) observational studies (case-control, cohort, or cross-sectional); 2) studies should include patients diagnosed with hip or knee OA and a group of people with no OA; 3) falls or fractures evaluation, and sufficient data on adjusted risks of falls, recurrent falls, and fractures between OA and non-OA groups (e.g. odds ratio (OR), risk ratio (RR), hazard ratio (HR)). Exclusion criteria included: 1) studies focus on topics irrelevant to our research interest; 2) no control group; 3) patients with diseases other than OA; 4) full text not available; and 5) no data available. There was no restriction on language. During the study selection process, we found that some studies evaluated the association of OA with falls, recurrent falls, or fractures using the same population. In such cases, we selected the study with the largest sample size for the outcomes of interest. This was not included in the registered protocol.

### Data extraction

Two authors (YZ and YW) independently extracted data from each included study. The extracted data included the first author, year of publication, place (country and continent), number of participants, follow-up time, OA sites, OA diagnoses (i.e. radiographic, symptomatic, self-reported, or clinician-diagnosed), reported outcomes measure (i.e. falls, recurrent falls and/or fractures), adjusted OR, RR,or HR, with 95% confidence interval (CI).

OA was divided into four groups according to its sites or diagnoses, namely, radiographic knee OA, radiographic hip OA, symptomatic knee OA, and symptomatic hip OA. Self-reported and clinician-diagnosed OA, and OA retrieved from medical records (e.g. the International Classification of Diseases, 9^th^ or 10^th^ revision (ICD-9 or ICD-10) [27]) were considered symptomatic OA because pain symptom is the leading cause of seeking medical attention [28], and the diagnosis of clinical OA for the knee and the hip, based on the American College of Rheumatology criteria (ACR) [29, 30], requires the presence of knee or hip pain but not radiographic evidence. For studies that included patients with knee OA and hip OA, data were extracted and analyzed separately to evaluate the associations of knee OA and hip OA with falls, recurrent falls, and fractures. Similarly, data from studies that reported both single and multiple falls were analyzed separately for falls and recurrent falls. For studies that reported both vertebral and non-vertebral fractures as the outcome measures, we pooled non-vertebral fractures for this study and conducted a post-hoc analysis for vertebral fractures as this was not documented in the registered protocol.

### Risk of bias and quality of evidence

Two authors (YZ and LG) independently assessed the risk of bias of included studies using the Newcastle-Ottawa Scale (NOS) [31]. The NOS was designed to assess the quality of the non-randomized study, and studies with a NOS score of < 7 were considered high risk of bias, and < 4 very high risk of bias [32]. Any disagreement was discussed with a third author (GC). For each outcome, the quality of evidence was assessed using the GRADE (Grading of Recommendations, Assessment, Development, and Evaluation) approach, which combines risk of bias, consistency of effect, imprecision, indirectness, and publication bias [33]. The quality of the evidence was downgraded from high to very low depending on the severity of each component in the GRADE. The GRADE Summary of Findings table was generated using the GRADEpro Guideline Development Tool on the GRADEpro website (https://www.gradepro.org/).

### Data synthesis

The summary measures used in this meta-analysis were confounder-adjusted OR, RR or HR. Before we pooled the data, OR, RR and HR were transformed into their natural logarithms [34] in order to stabilize the variance and normalize the distribution. We derived natural logarithm variance of OR, HR and RR from their corresponding 95% CIs provided in the original reports. In accordance with the Cochrane handbook [35], we changed our protocol in PROSPERO and no longer chose fixed- or random-effects model based on heterogeneity of the pooled effect, but used a random-effects model (Der Simonian and Laird) [36] uniformly to calculate pooled effects and 95% CIs for the association of radiographic and symptomatic knee and hip OA with the risk of falls, recurrent falls, and fractures.

Statistical analyses were performed with R statistical software (Version 4.1.3).

### Assessment of heterogeneity

We used both the Q and the I^2^ statistics to test the homogeneity of effect size, where *P* < 0.05 and I^2^ > 50% was considered heterogeneous.

### Sensitivity analysis

Sensitivity analysis was conducted to examine the influence of omitting case-control and cross-sectional studies on the pooling results. Another sensitivity analysis was conducted by excluding studies in which falls and fractures were self-reported.

### Assessment of publication bias

Due to the limited number of studies included in this meta-analysis, we only used funnel plots to visually assess publication bias. The Begg’ test indicated in the registered protocol was not performed.

## Results

### Study selection

**Fig. 1:**
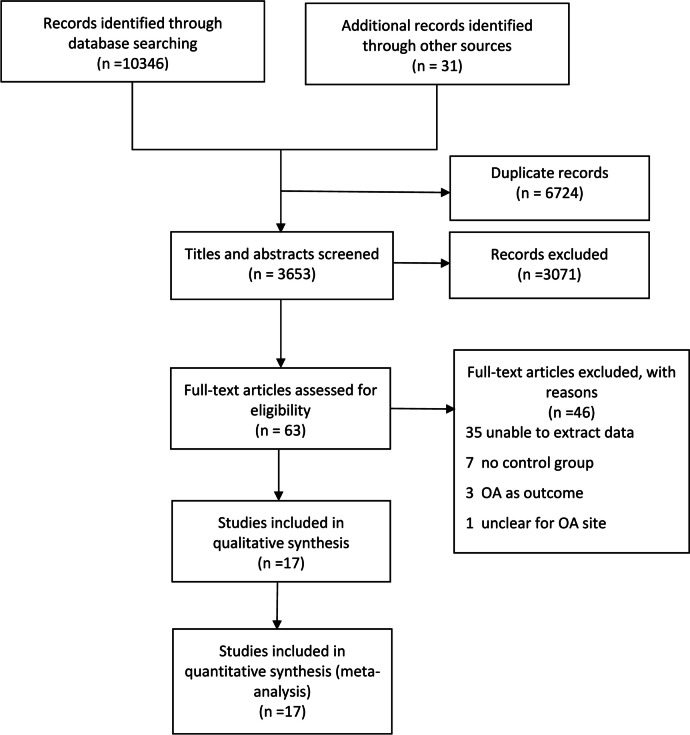
Study flowchart

Figure 1 describes the flow chart of the study selection process. We identified 10377 potentially relevant publications from electronic searching. Of these, 6724 were excluded due to duplication, and 3071 were excluded after reviewing the titles and abstracts. Forty-six studies were excluded after full-text review: 36 studies did not provide sufficient data for the meta-analysis, 7 did not have a control group, 3 did not treat falls or fractures as an outcome measure, and 1 did not specify OA site. Finally, we included 17 studies with 862849 participants in the systematic review.

### Study characteristics and quality assessment

Table 1 summarizes the main characteristics of the 17 included studies. The majority were cohort studies (*n* = 13), 1 was cross-sectional and 3 were case-control studies. The follow-up period of the cohort studies ranged from 1 to 28 years. Among the 17 studies included, 10 reported symptomatic OA, 9 reported radiographic OA, and only 2 reported both. Symptomatic OA was self-reported in 6 of the 10 studies, and the remaining 4 were defined based on medical records (i.e. ICD-10, ACR criteria) [37–40]. All studies reported falls based on self-reported records (in the past 4-12 months), except one using medical records (i.e. ICD-9) [40]. Fractures were self-reported in 5 studies [16, 19, 39, 41, 42], and the remaining were based on medical records (i.e. hospital database, ICD-10 or ICD-9) [14, 17, 18, 37, 40, 43]. Eight studies included both knee and hip OA patients, 2 studies reported both falls and fractures outcomes, and 3 studies reported both falls and recurrent falls outcomes. Two studies reported both non-vertebral and vertebral fractures. One study was not evaluated for NOS score because it is a conference abstract [39]. Two studies had NOS scores of < 7 and were considered to have a high risk of bias (Supplementary Table 1).

**Table 1:** Characteristics of the individual studies included in this systematic review and meta-analysis

**Author**	**Place**	**Population**	**Study design**	**Follow-up**	**OA site and type**	**Outcomes**^**a**^	**Variables in adjusted models**
**Usova et al. 2022 **[39]	Russia	*N* = 98, 100% women, median age 63.0 [59.3;69.8] years	Cohort	-	Symptomatic knee	Fractures	-
**Iijima et al. 2021 **[25]	Japan	*N* = 291, 78.7% women, ≥ 60 years	Cross-sectional	-	Radiographic knee	Single fall, Recurrent falls	Age, sex, BMI
**Jacob et al. 2021 **[37]	UK	*N* = 258,696,60.1% women, (63.7 ± 14.0) years	Cohort	10 years	Symptomatic knee Symptomatic hip	Fractures	Age, sex, index year, diabetes, dementia, and corticosteroid use
**Soh et al. 2020 **[16]	US	*N* = 4796,58.5% women, ≥ 45 years	Cohort	8 years	Symptomatic knee^b^ Symptomatic hip^b^	Falls, Fractures	Age, gender, previous history of falls, depression, living situation, extensor strength, mobility limitations, difficulties with sport and recreation
**Van Schoor et al. 2020 **[10]	Europe	*N* = 2535, 65–85 years	Cohort	1 year	Symptomatic knee^b^ Symptomatic hip^b^	Falls, Recurrent falls	Age, sex, country, education, BMI, alcohol use, number of chronic diseases
**Barbour et al. 2018 **[40]	US	*N* = 734, 33.2% women, 70–79 years	Cohort	6.5 years	Radiographic knee Symptomatic knee	Falls	Age, sex, race, education, BMI, physical activity, smoking, health status, history of falls, diabetes, hypertension, myocardial infarction, medications
**Bergink et al. 2018 **[41]	Netherlands	*N* = 3005, 55.8% women, 65.3 ± 6.5 years	Cohort	8.4 years	Radiographic knee Radiographic hip	Fractures	Age, sex, BMI, femoral neck BMD, use of walking aid, lower limb disability, fall tendency
**Dore et al. 2015 **[13]	US	*N* = 1619, 67.3% women, ≥ 45 years	Cohort	3–10 years	Symptomatic knee^b^ Symptomatic hip^b^	Falls	Age, BMI, sex, race, history of falls
**Rouzi et al. 2015 **[9]	Saudi Arabia	*N* = 707, 100% women, 61.33 ± 7.19 years	Cohort	5.2 years	Radiographic knee	Single fall, Recurrent falls	Age, BMI, knee pain, lower back pain, vitamin D status
**Yamamoto et al. 2015 **[19]	Sweden	*N* = 74,598 52% women, ≥ 45 years	Cohort	4 years	Symptomatic knee^b^ Symptomatic hip^b^	Fractures	-
**Muraki et al. 2013 **[44]	Japan	*N* = 2215, 66.4% women, mean70.6 years	Cohort	3 years	Radiographic knee	Falls,	Age, BMI
**Castaño-Betancourt et al. 2013 **[15]	Netherlands	*N* = 5006, 56.7% women, ≥ 55 years	Cohort	9.6 years	Radiographic hip	Osteoporotic fractures	Age, sex, height, weight, BMD
**Franklin et al. 2011 **[18]	Iceland	*N* = 2953, 57% women, ≥ 35 years	Cohort	11–28 years	Radiographic hip	Fractures	Age, sex
**Vestergaard et al. 2009 **[38]	Denmark	*N* = 498,617,51.8% women (43.44 ± 27.39) years	case–control	-	Symptomatic knee Symptomatic hip	Fractures	Prior fracture, alcoholism, Charlson index, use of corticosteroids, use of drugs against epilepsy, use of diuretics, income, living alone or not, working status, use of strong analgesics, use of weak analgesics, number of bed days and number of contacts to general practitioner or specialist
**Arden et al. 1999 **[17]	US	*N* = 5552, 100% women, 71.4 ± 5.1 years	Cohort	7.4 years	Radiographic hip Symptomatic hip^b^	Recurrent falls, Osteoporotic fractures	Age, knee height, weight at age 50, clinical centers
**Arden et al. 1996 **[14]	UK	*N* = 939, 100% women, ≥ 45 years	Case–control	-	Radiographic knee Radiographic hip	Osteoporotic fractures	Age, weight, oral contraceptive pill usage, hormone replacement therapy usage, menopausal status, menopause duration, smoking and alcohol use, BMD, sex hormones
**Cumming et al. 1993 **[43]	Australia	*N* = 416, 35.8% women, ≥ 65 years	Case–control	-	Symptomatic knee^b^ Symptomatic hip^b^	Fractures	Age, sex, BMI, current physical activity, and occupational physical activity at age 50 years

*OA* osteoarthritis, *BMI* body mass index, *BMD* bone mineral density

^a^Recurrent falls were defined as two or more falls in the past year in all studies

^b^self-reported OA

### Knee OA and falls, recurrent falls, fractures

**Fig. 2:**
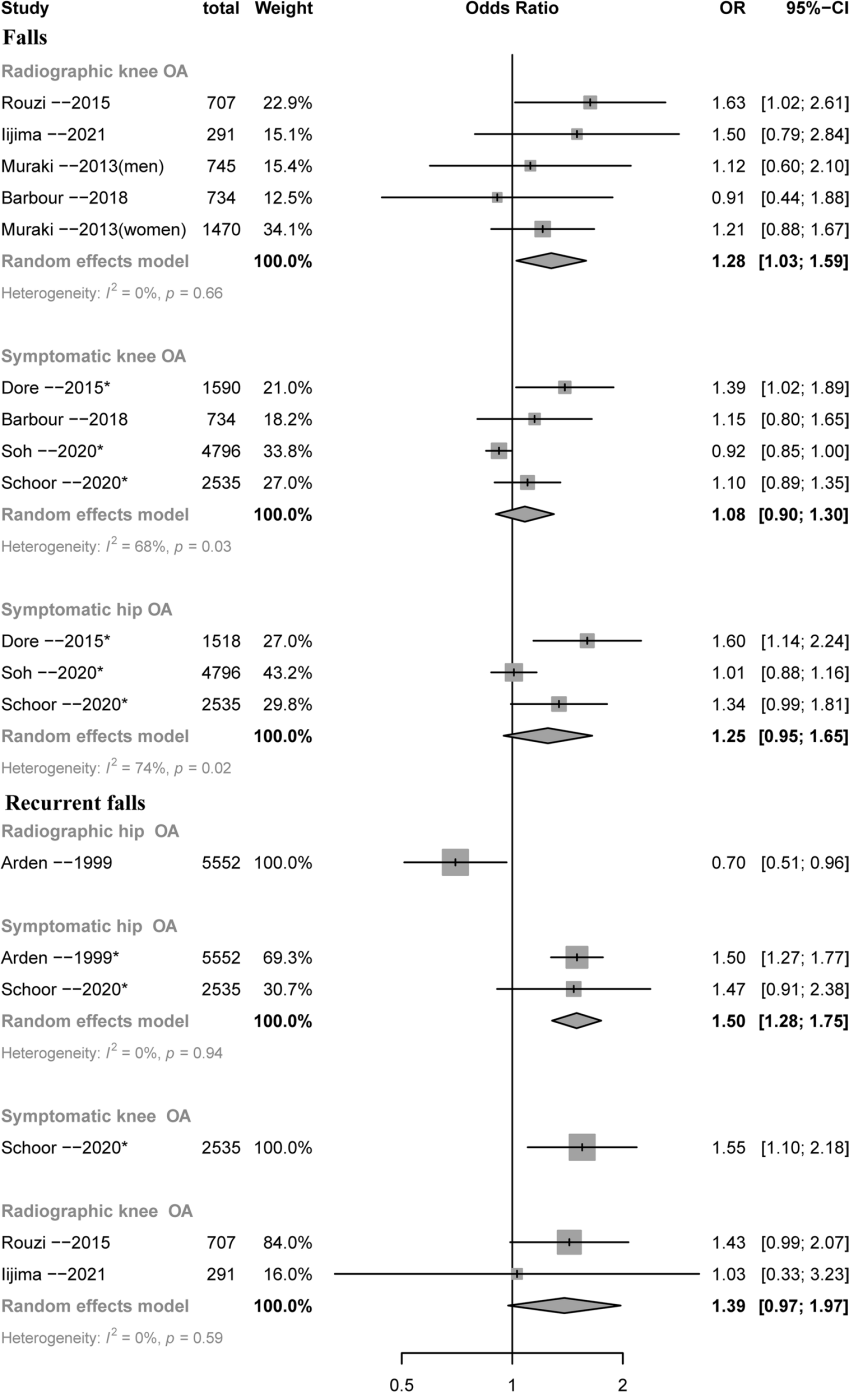
Forest plot for the associations of knee and hip osteoarthritis with falls and recurrent falls. *Self-reported OA

**Fig. 3:**
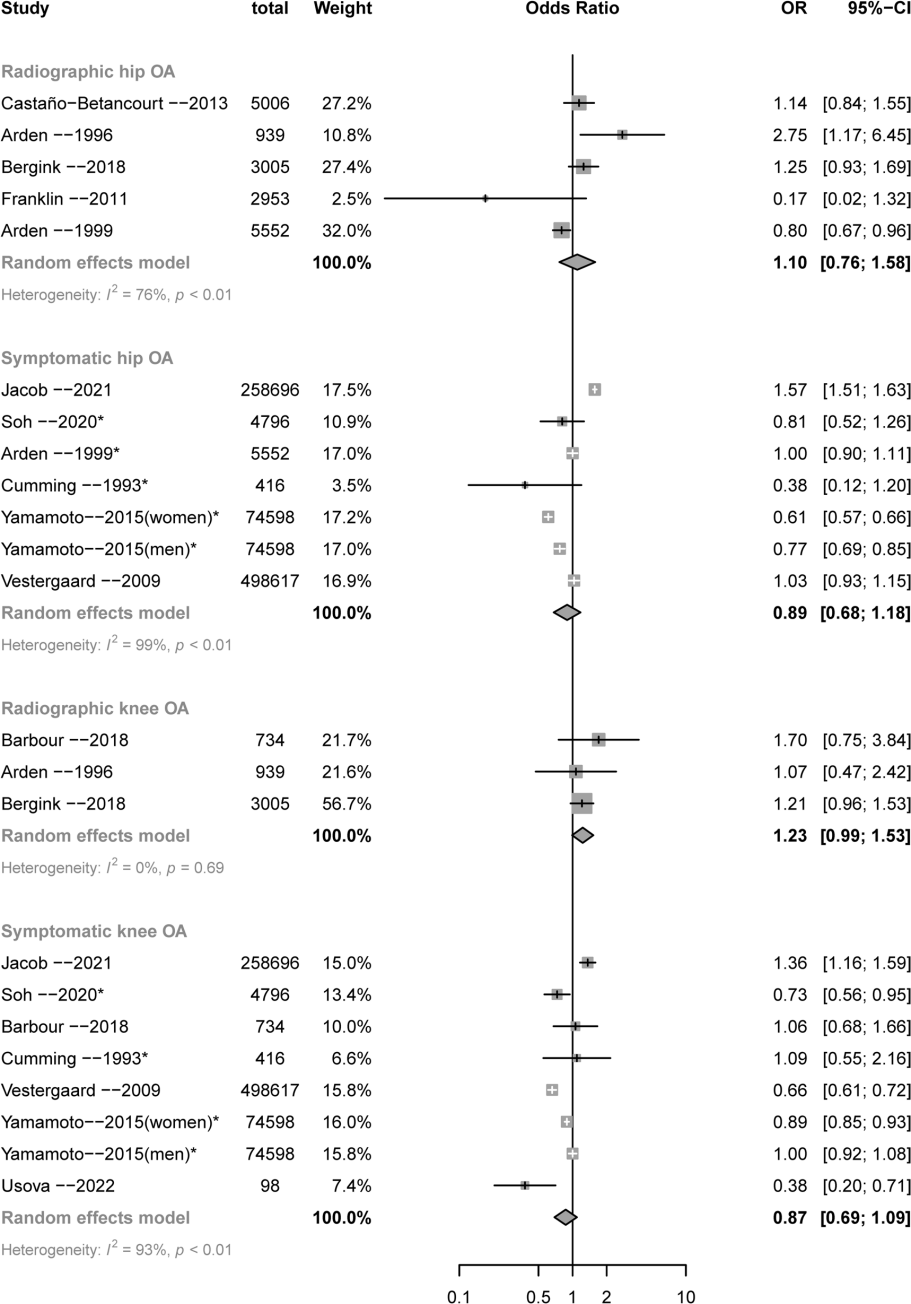
Forest plot for the associations of knee and hip osteoarthritis with fractures. *Self-reported OA

Table 2 and Figs. 2 and 3 summarizes the associations of symptomatic and radiographic knee OA with the risk of falls, recurrent falls, and fractures. Symptomatic knee OA was associated with increased risk of recurrent falls (OR = 1.55, 95% CI 1.10 to 2.18, 1 study, n = 2535 from 1 study, GRADE: High) but not falls or any fractures (Table 2 and Fig. 3). Radiographic knee OA was associated with increased risk of falls (OR = 1.28, 95% CI 1.03 to 1.59, *n* = 3947 from 4 studies, I^2^ = 0%, GRADE: Moderate), and showed no statistically significant association with recurrent falls (OR = 1.39, 95% CI 0.97 to 1.97, *n* = 998 from 2 studies, I^2^ = 0%, GRADE: Low) and fractures (OR = 1.22, 95% CI 0.99 to 1.52, *n* = 4678 from 3 studies, I^2^ = 0%, GRADE: Low).

**Table 2:** Association of radiographic and symptomatic knee osteoarthritis with the risks of falls, recurrent falls, and fractures

**Outcomes**	**Odds ratio (95% CI)**	**No. of participants (studies)**	**Certainty of the evidence (GRADE)**	**Comments**
*Falls*				
SOA	1.08 (0.90 to 1.30)	9635 (4 studies)	Low (inconsistency and publication bias)	SOA may have no effect for increasing risk of falls
ROA	**1.28 (1.03 to 1.59)**	3927 (4 studies)	Moderate (publication bias)	ROA may have a very small effect for increasing risk of falls
*Recurrent falls*				
SOA	**1.55 (1.10 to 2.18)**	2535 (1 study)	Moderate (imprecision)	SOA may have a small effect for increasing risk of recurrent falls
ROA	1.39 (0.97 to 1.97)	998 (2 studies)	Low (imprecision and publication bias)	ROA may have very small or no effect for increasing risk of recurrent falls
*Fractures*				
SOA	0.87 (0.69 to 1.09)	912553 (7 studies)	Very low (risk of bias, inconsistency and publication bias)	SOA may have no effect for reducing risk of fractures
ROA	1.23 (0.99 to 1.53)	4678 (3 studies)	Low (risk of bias and publication bias)	ROA may have no effect for reducing risk of fractures

Risk of bias: The case group definition for Cumming et.al [43] is based on self-report, with selection bias and no response rate not stated. Case group definition for Arden et.al [14] is based on self-report, with selection bias and control group selected from hospital. Inconsistency: downgraded because the proportion of variance in the effect estimates caused by true heterogeneity rather than chance is important (I^2^ > 50%); Publication bias: funnel plot indicates a potential publication bias. Imprecision: only 1 study was available, and the wide CI may influence clinical decision

*CI* confidence interval, *GRADE* Grading of Recommendations, Assessment, Development, and Evaluation, *ROA* radiographic osteoarthritis, *SOA* symptomatic osteoarthritis

### Hip OA and falls, recurrent falls, fractures

Table 2 and Fig. 2 and 3 summarizes the associations of symptomatic and radiographic hip OA with the risk of falls, recurrent falls, and fractures. Symptomatic hip OA was associated with increased risk of recurrent falls (OR = 1.50, 95% CI 1.28 to 1.75, *n* = 8087 from 2 studies, I^2^ = 0%, GRADE: Moderate) and showed no statistically significant association with falls (OR = 1.25, 95% CI 0.95 to 1.65, *n* = 8849 from 3 studies, I^2^ = 74%, GRADE: Low). No study evaluated the association of radiographic hip OA with risk of falls, but in one study radiographic hip OA was associated with a lower risk of recurrent falls (OR = 0.70, 95% CI 0.51 to 0.96, *n* = 5552 from 1 study, GRADE: High, Fig. 2). There were no statistically significant associations between either symptomatic or radiographic hip OA with fractures (Table 3 and Fig. 3).

**Table 3:** Association of radiographic and symptomatic hip osteoarthritis with the risks of falls, recurrent falls, and fractures

**Outcomes**	**Odds ratio (95% CI)**	**No. of participants (studies)**	**Certainty of the evidence (GRADE)**	**Comments**
*Falls*				
SOA	1.25 (0.95 to 1.65)	8849 (3 studies)	Low (inconsistency and publication bias)	SOA may have very small or no effect for increasing risk of falls
*Recurrent falls*				
SOA	**1.50 (1.28 to 1.75)**	8087 (2 study)	Moderate (publication bias)	SOA may have a small effect for increasing risk of recurrent falls
ROA	**0.70 (0.50 to 0.95)**	5552 (1 study)	Moderate (imprecision)	ROA may have a very small effect for reducing risk of recurrent falls
*Fractures*				
SOA	0.89 (0.68 to 1.18)	917273(6 studies)	Very low (risk of bias, inconsistency, and publication bias)	SOA may have no effect for reducing risk of fractures
ROA	1.10 (0.76 to 1.58)	17,455 (5 studies)	Very low(risk of bias, inconsistency, and publication bias)	ROA may have no effect for increasing risk of fractures

Risk of bias: The case group definition for Cumming et.al [43] is based on self-report, with selection bias and no response rate not stated. Case group definition for Arden et.al [14] is based on self-report, with selection bias and control group selected from hospital. Inconsistency: downgraded because the proportion of variance in the effect estimates caused by true heterogeneity rather than chance is important (I^2^ > 50%); Publication bias: funnel plot + indicates a potential publication bias. Imprecision: only 1 study was available, and the wide CI may influence clinical decision

*CI* confidence interval, *GRADE* Grading of Recommendations, Assessment, Development, and Evaluation, *ROA* radiographic osteoarthritis, *SOA* symptomatic osteoarthritis

### Sensitivity analysis

The omission of case-control and cross-sectional studies did not substantially alter the primary outcome (Supplementary Tables S2 and S3), except that the association between radiographic knee OA and risk of recurrent falls became statistically significant (OR = 1.43, 95% CI 1.01 to 2.12, *n* = 707 from 1 study, GRADE: Moderate). The exclusion of studies using self-reported falls and fractures did not change the main findings (Supplementary Tables S4 and S5), except that the associations between radiographic knee OA with fractures became statistically significant (OR = 1.34, 95% CI 1.15 to 1.57, *n* = 259,112 from 2 studies, GRADE: low).

### Publication bias

Visual inspection of the funnel plots showed possible asymmetry for the associations of knee and hip OA with falls, recurrent falls, and fractures (Supplementary Figures S1 and S2).

### Post-hoc analysis

There was no statistically significant association of radiographic hip and knee OA and symptomatic hip OA with the risk of vertebral fractures (Table S6).

## Discussion

In this systematic review and meta-analysis of 17 observational studies, we evaluated the association of radiographic and symptomatic knee and hip OA in the risk of falls and fractures. The pooled results revealed that both symptomatic knee and hip OA were associated with an increased risk of recurrent falls, but that neither symptomatic knee nor hip OA was associated with the risk of fractures. Radiographic knee OA was associated with an increased risk of falls. Overall, the quality of evidence of included studies was moderate to very low, but nonetheless symptomatic knee and hip should be considered as potential risk factors for falls and falls risk assessment and preventive interventions in people with OA at these sites considered.

While only radiographic knee OA was associated with an increased risk of falls, both symptomatic and radiographic knee OA showed a similar magnitude in the increased risk of recurrent falls, though for radiographic knee OA this was not statistically significant. This is likely to reflect a real effect of knee OA on falls because a single fall may be coincidental, whereas recurrent falls are more likely to have an internal, disease-related cause [10]. Recurrent falls generally lead to more serious consequences, such as prolonged hospitalization, immobility and even death, compared to single fall events [45]. Considering the high prevalence of knee OA and the severity of falls in older adults, it is important to strengthen medical care and develop preventive interventions to reduce falls in this population. In patients with knee and hip OA, routine use of screening tools may help to identify those at increased risks of falls and fractures [46], and this is likely to promote the implementation of primary preventions, such as self-management, home safety resources, and more intensive clinical care [47]. Existing evidence has shown that exercise programs, such as strength training, tai chi, and aerobic exercises, can strengthen the muscles of the lower limb and improve balance, and thus reducing the risk of falls and the fear of falling in people with OA [48], and could be considered for people with knee and hip OA. However, OA patients are different from the general population in many aspects, including but not limited to joint stability, balance, muscle strength, and bone quality; therefore, more research should be performed to evaluate whether OA-specific screening tools could provide a more precise estimation for falls and fractures.

Despite falls being a risk factor for fractures, and symptomatic knee and hip OA being associated with falls, there were no statistically significant associations between symptomatic knee or hip OA and the risk of any fractures or of vertebral fractures. However, in a recent study, we found that the associations of bilateral knee symptoms with the risk of fractures were attenuated and no longer statistically significant after further adjusting for falls [11], suggesting that falls could mediate effects of knee symptoms on fractures. This finding is consistent with our sensitivity analysis showing that symptomatic knee OA was associated with an increased risk of fractures after removing studies that recorded self-reported falls and fractures. Thus, the potential role of knee OA in fractures cannot be ruled out.

While symptomatic hip OA was associated with an increased risk of recurrent falls, radiographic hip OA was associated with a decreased risk. These findings came from a single study of 939 older women [14] and contrast to our findings that both symptomatic and radiographic knee OA increased the risk of recurrent falls. The reasons for such a protective effect are unclear and further studies are needed to determine the associations of radiographic knee and hip OA with fracture risk. It remains unclear whether sex has a modification effect on the association between OA and fracture risk. In this meta-analysis, only 1 study analyzed the association between OA and fracture risk by sex and showed no significant difference [19]. In a post-hoc study of a randomized controlled trial, women with knee pain or clinician-diagnosed knee OA were found to have a higher risk of fractures, but it is unknown whether the association between knee OA and fracture risk was stronger in women [42]. In our recent study using data from the Osteoarthritis Initiative, however, we found that men but not women with unilateral knee symptoms had a higher risk of fractures [11]. Therefore, the role of sex in the association between OA and fracture risk needs further study.

A recent meta-analysis evaluated the association of knee and hip OA with the risk of falls [20], but unlike our review, it analyzed self-reported and radiographic OA in pooling data without separating it into self-reported and symptomatic knee or hip osteoarthritis. This study found that knee but not hip OA was positively associated with the risk of falls (RR: 1.46; *P* < 0.01), and that radiographic OA (knee and hip OA combined) was not significantly associated with fall risk (*P* > 0.05) [20]. This pooling of OA sites and of both symptomatic and radiological OA may be problematic given there are significant differences between knee and hip OA [21]. For example, the experience of pain is different between hip OA and knee OA [21], instability in hip OA may be more likely to occur than instability in knee OA [22]. Moreover, there is discordance between radiographic and symptomatic OA [23, 24]. Indeed, when we updated the literature search and separated symptomatic and radiographic OA for both the knee and the hip, we found that while symptomatic knee and hip OA were both associated with an increased risk of recurrent falls (OR = 1.28, *P* < 0.05), radiographic knee OA was associate with an increased and radiographic hip OA with a decreased risk of falls (OR = 0.70, *P* < 0.05). More studies are needed in the future to examine our findings.

This systematic review was carried out following a pre-specified registered protocol and reported using the PRISMA checklist. We described in detail the associations of radiographic and symptomatic OA of the knee and hip with falls, recurrent falls, and fractures using adjusted results, which are more reflective of the true association. However, there were several limitations in the study. First, the quality of the evidence was moderate to very low due to the large differences between enrolled studies in terms of study design, population characteristics, time of follow-up, definitions of OA, and adjudication of falls and fractures. Moreover, some pooled results were only based on one or two individual studies, making the results less convincing. Nonetheless, this meta-analysis provides the most robust currently available evidence addressing this important question. Second, data on falls and fractures are mostly self-reported and may have been subjected to recall biases. Prospective and objective data on falls and fractures would be preferred. Third, publication bias was indicated by the asymmetry funnel plots, although we have searched and screened gray literature such as conference abstracts. Fourth, while we restricted to adjusted data in the pooled results, the variables adjusted were different among studies. This could be resolved by conducting an individual participant data meta-analysis. Moreover, in consideration of the dissimilar effects of radiographic and symptomatic OA on especially fracture risks, simultaneously evaluating the association of both symptomatic and radiographic definitions of OA with the risk of falls and fractures in the same study population is recommended in future studies.

## Conclusions

Symptomatic knee and hip OA were both associated with an increased risk of recurrent falls, and radiographic knee OA was associated with an increased risk of falls. However, we did not find statistically significant associations of radiographic and symptomatic knee or hip OA with fractures. Symptomatic knee and hip should be considered as potential risk factors for falls and falls risk assessment and preventive interventions in people with OA at these sites considered.

### Supplementary Information

**Additional file 1: Supplemental Methods.** Search strategy. **Table S1.** The methodological quality of included studies in accordance with the Newcastle-Ottawa Scale (NOS). **Table S2.** Association of radiographic and symptomatic knee osteoarthritis with falls, recurrent falls, and fractures*. **Table S3.** Association of radiographic and symptomatic hip osteoarthritis with falls, recurrent falls and fractures*. **Table S4.** Association of radiographic and symptomatic knee osteoarthritis with falls, recurrent falls and fractures*. **Table S5.** Associations of radiographic and symptomatic hip osteoarthritis with falls, recurrent falls and fractures*. **Table S6.** Evaluating the effect of radiographic and symptomatic hip and radiographic osteoarthritis on vertebral fractures. **Figure S1.** Funnel plots for the associations of radiographic and symptomatic knee osteoarthritis with falls and fractures. a: radiographic knee osteoarthritis and falls; b: symptomatic knee osteoarthritis and fractures. **Figure S2.** Funnel plots for the associations of radiographic and symptomatic hip osteoarthritis with falls and fractures. c: symptomatic knee osteoarthritis and falls; d: radiographic knee osteoarthritis and fractures.

## Data Availability

The data analyzed during the current study are available from the corresponding author (GC) upon reasonable request.

## Abbreviations

OA: Osteoarthritis
ICD: The International Classification of Diseases
ACR: American College of Rheumatology criteria
NOS: Newcastle-Ottawa Scale
GRADE: Grading of Recommendations, Assessment, Development, and Evaluation
SOA: Symptomatic Osteoarthritis
ROA: Radiographic Osteoarthritis
