# Supplementary material for: Association of knee and hip osteoarthritis with the risk of falls and fractures: a systematic review and meta-analysis
Source: Arthritis Res Ther. 2023 Sep 29;25:184. doi: 10.1186/s13075-023-03179-4 (PMC10540335; doi:10.1186/s13075-023-03179-4)
Supplement: Supplementary file 1 — Additional file 1: Supplemental Methods. Search strategy. Table S1. The methodological quality of included studies in accordance with the Newcastle-Ottawa Scale (NOS). Table S2. Association of radiographic and symptomatic knee osteoarthritis with falls, recurrent falls, and fractures*. Table S3. Association of radiographic and symptomatic hip osteoarthritis with falls, recurrent falls and fractures*. Table S4. Association of radiographic and symptomatic knee osteoarthritis with falls, recurrent falls and fractures*. Table S5. Associations of radiographic and symptomatic hip osteoarthritis with falls, recurrent falls and fractures*. Table S6. Evaluating the effect of radiographic and symptomatic hip and radiographic osteoarthritis on vertebral fractures. Figure S1. Funnel plots for the associations of radiographic and symptomatic knee osteoarthritis with falls and fractures. a: radiographic knee osteoarthritis and falls; b: symptomatic knee osteoarthritis and fractures. Figure S2. Funnel plots for the associations of radiographic and symptomatic hip osteoarthritis with falls and fractures. c: symptomatic knee osteoarthritis and falls; d: radiographic knee osteoarthritis and fractures. [file 13075_2023_3179_MOESM1_ESM.docx]

# **Supplemental Methods – Search strategy**

**Medline**

1. Accidental Falls/

2. (fall or falls).tw.

3. exp Osteoarthritis/

4. (arthrit$ or osteoarthrit$).ti,ab.

5. 1 or 2

6. 3 or 4

7. Fracture Healing/

8. exp Fracture Fixation/

9. exp Fractures, Bone/

10. fracture*.tw.

11. 7 or 8 or 9 or 10

12. 5 or 11

13. 6 and 12

14. exp Animals/ not Humans/

15. Epidemiologic Studies/

16. exp Cohort Studies/

17. exp Case Control Studies/

18. Cross Sectional Studies/

19. (epidemiologic adj (study or studies)).ab,ti.

20. case control.ab,ti.

21. (cohort adj (study or studies)).ab,ti.

22. cross sectional.ab,ti.

23. cohort analy$.ab,ti.

24. (follow up adj (study or studies)).ab,ti.

25. longitudinal.ab,ti.

26. retrospective$.ab,ti.

27. prospective$.ab,ti.

28. (observ$ adj3 (study or studies)).ab,ti.

29. 15 or 16 or 17 or 18 or 19 or 20 or 21 or 22 or 23 or 24 or 25 or 26 or 27 or 28

30. 29 not 14

31. 13 and 30

32. knee$.tw.

33. exp Knee Joint/

34. Knee/

35. 32 or 33 or 34

36. 6 and 35

37. 12 and 30 and 36

38. Hip/

39. exp Hip Joint/

40. hip$.tw.

41. 38 or 39 or 40

42. 35 or 41

43. 6 and 42

44. 12 and 30 and 43

**Embase**

1 hip$.tw.

2 Hip/

3 arthrosis.tw.

4 (degenerative adj2 arthritis).tw.

5 osteoarthr$.tw.

6 exp osteoarthritis/

7 (fall or falls).tw.

8 Accidental Falls/

9 exp Fracture/

10 fracture*.tw.

11 Falling/

12 epidemiology/

13 exp case control study/

14 cohort analysis/

15 cross sectional study/

16 case study/

17 follow up/

18 longitudinal study/

19 retrospective study/

20 prospective study/

21 observational study/

22 correlational study/

23 epidemiologic.ab,ti.

24 case control.ab,ti.

25 case referent.ab,ti.

26 case stud$.ab,ti.

27 case series.ab,ti.

28 cohort?.ab,ti.

29 cross sectional.ab,ti.

30 follow up.ab,ti.

31 longitudinal.ab,ti.

32 retrospective$.ab,ti.

33 prospective$.ab,ti.

34 observational.ab,ti.

35 (Controlled before and after).ab,ti.

36 Interrupted time series.ab,ti.

37 Correlational.ab,ti.

38 ecological stud$.ab,ti.

39 Descriptive stud$.ab,ti.

40 Knee/

41 knee$.tw.

42 1 or 2

43 3 or 4 or 5 or 6

44 7 or 8 or 11

45 9 or 10

46 44 or 45

47 40 or 41

48 42 or 47

49 12 or 13 or 14 or 15 or 16 or 17 or 18 or 19 or 20 or 21 or 22 or 23 or 24 or 25 or

26 or 27 or 28 or 29 or 30 or 31 or 32 or 33 or 34 or 35 or 36 or 37 or 38 or 39

50 exp Animals/ not Humans/

51 49 not 50

52 43 and 46 and 48 and 51

**Web of science**

1 TI= ( falls or faller* ) OR AB= ( falls or faller* )

2 TI= ( falls or faller* ) OR AB= ( falls or faller* ) OR TS=( falls or faller* )

3 TI= fractur* OR AB= fractur* OR TS= fractur*

4 TI= knee* OR AB= knee* OR TS=knee*

5 TI=osteoarthr* OR AB= osteoarthr* OR TS=osteoarthr*

6 TI=arthrosis OR AB=arthrosis OR TS=arthrosis

7 TI=(degenerative next arthritis) OR AB=(degenerative next arthritis) OR

TS=(degenerative next arthritis)

8 TI= hip* OR AB= hip* OR TS=hip*

9 (TS= "Case Control Studies+")

10 (TS= "Case Control Studies+")OR(TS= "Case Control Studies+")OR (TS= "Case

Studies")OR (TS= "Cross Sectional Studies")OR (TS= "Prospective Studies+")OR

(TS= "Retrospective Panel Studies")OR (TS= "Correlational Studies")OR (TS=

"Ecological Research")OR (TS= "Descriptive Research")

11 TI= epidemiologic OR AB =epidemiologic

12 TI ="case control" OR AB="case control"

13 TI =“case referent” OR AB=“case referent”

14 TI =“case stud*” OR AB=“case stud*”

15 TI = “case series” OR AB= “case series”

16 TI = cohort* OR AB= cohort*

17 TI = “cross sectional” OR AB= “cross sectional”

18 TI = “follow up” OR AB= “follow up”

19 TI = longitudinal OR AB= longitudinal

20 TI =retrospective* OR AB= retrospective*

21 TI = prospective* OR AB= prospective*

22 TI =observational OR AB= observational

23 TI =“Controlled before and after” OR AB= “Controlled before and after”

24 TI =“Interrupted time series” OR AB= “Interrupted time series”

25 TI =Correlational OR AB= Correlational

26 TI= “ecological stud*” OR AB= “ecological stud*”

27 TI= “Descriptive stud*” OR AB =“Descriptive stud*”

28 #27 OR #26 OR #25 OR #24 OR #23 OR #22 OR #21 OR #20 OR #19 OR #18

OR #17 OR #16 OR #15 OR #14 OR #13 OR #12 OR #11 OR #10

29 #7 OR #6 OR #5

30 #4 OR #8

31 #30 AND #29

32 #2 OR #3

33 #32 AND #31 AND #28

# **Supplementary Tables**

Table S1. The methodological quality of included studies in accordance with the Newcastle-Ottawa Scale (NOS).

| **Authors** | **Year** | **Selection** | | | | **Comparability** | **Outcome/exposure *** | | | **Overall** |
| --- | --- | --- | --- | --- | --- | --- | --- | --- | --- | --- |
|  |  | **1** | **2** | **3** | **4** |  | **1** | **2** | **3** |  |
| Barbour et.al | 2018 | 1 | 1 | 1 | 0 | 2 | 1 | 1 | 0 | 7 |
| Jacob et.al | 2021 | 1 | 1 | 1 | 0 | 2 | 1 | 1 | 0 | 7 |
| Bergink et.al | 2018 | 1 | 1 | 1 | 0 | 2 | 1 | 1 | 0 | 7 |
| Dore et.al | 2015 | 1 | 1 | 1 | 0 | 2 | 1 | 1 | 0 | 7 |
| Rouzi et.al | 2015 | 1 | 1 | 1 | 0 | 2 | 1 | 1 | 0 | 7 |
| Yamamoto et.al | 2015 | 1 | 1 | 1 | 0 | 2 | 1 | 1 | 0 | 7 |
| Muraki et.al | 2013 | 1 | 1 | 1 | 1 | 2 | 1 | 1 | 0 | 8 |
| Castaño-Betancourt et.al | 2013 | 1 | 1 | 1 | 0 | 2 | 1 | 1 | 0 | 7 |
| Franklin et.al | 2011 | 1 | 1 | 1 | 0 | 2 | 1 | 1 | 0 | 7 |
| Arden et.al | 1999 | 1 | 1 | 1 | 0 | 2 | 1 | 1 | 0 | 7 |
| Soh et.al | 2020 | 1 | 1 | 1 | 0 | 2 | 1 | 1 | 0 | 7 |
| Schoor et.al | 2020 | 1 | 1 | 1 | 1 | 2 | 0 | 1 | 0 | 7 |
| Iijima et.al | 2021 | 1 | 0 | 1 | 1 | 2 | 1 | 1 | 0 | 7 |
| Vestergaard et.al | 2009 | 1 | 1 | 1 | 1 | 2 | 1 | 1 | 0 | 8 |
| Cumming et..al | 1993 | 0 | 0 | 1 | 1 | 2 | 1 | 1 | 0 | 6 |
| Arden et.al | 1996 | 0 | 0 | 0 | 1 | 2 | 1 | 1 | 1 | 6 |

*Outcome and exposure assessments were conducted for cohort studies and case-control studies, respectively.

Table S2. Association of radiographic and symptomatic knee osteoarthritis with falls, recurrent falls, and fractures. *

| **Outcomes** | **Odds ratio (95% CI)** | **№ of participants**  **(studies)** | **Certainty of the evidence**  **(GRADE)** | **Comments** |
| --- | --- | --- | --- | --- |
| *Falls* |  |  |  |  |
| SOA | 1.08 (0.90 to 1.30) | 9635 (4 studies) | Low (inconsistency and publication bias) | SOA may have no effect for increasing risk of falls |
| ROA | **1.28 (1.01 to 1.62)** | 3636 (3 studies) | Moderate (publication bias) | ROA may have a very small effect for increasing risk of falls |
| *Recurrent falls* |  |  |  |  |
| SOA | 1.55 (1.10 to 2.18) | 2535 (1 study) | Moderate (imprecision) | SOA may have a small effect for increasing risk of recurrent falls |
| ROA | **1.43 (1.01 to 2.12)** | 707 (1 study) | Moderate (imprecise) | ROA may have very small or no effect for increasing risk of recurrent falls |
| *Fractures* |  |  |  |  |
| SOA | 0.90 (0.68to 1.18) | 413520(5 studies) | Low (inconsistency, and publication bias) | SOA may have no effect for reducing risk of fractures |
| ROA | 1.24 (0.99 to 1.65) | 3739 (2 studies) | Low (inconsistency and publication) | ROA may have no effect for reducing risk of fractures |

*case-control and cross-sectional studies were removed.

CI: confidence interval; GRADE, Grading of Recommendations, Assessment, Development, and Evaluation. ROA: radiographic osteoarthritis; SOA: symptomatic osteoarthritis.

Inconsistency: downgraded because the proportion of variance in the effect estimates caused by true heterogeneity rather than chance is important (I^2^ >50%); Publication bias: funnel plot indicates a potential publication bias. Imprecision: only 1 study was available, and the wide CI may influence clinical decision.

Table S3. Association of radiographic and symptomatic hip osteoarthritis with falls, recurrent falls and fractures. *

| **Outcomes** | **Odds ratio (95% CI)** | **№ of participants**  **(studies)** | **Certainty of the evidence**  **(GRADE)** | **Comments** |
| --- | --- | --- | --- | --- |
| *Falls* |  |  |  |  |
| SOA | 1.25 (0.95 to 1.65) | 8849 (3 studies) | Low (inconsistency and publication bias) | SOA may have very small or no effect for increasing risk of falls |
| *Recurrent falls* |  |  |  |  |
| SOA | **1.50 (1.28 to 1.75)** | 8087 (2 study) | Moderate (publication bias) | SOA may have a small effect for increasing risk of recurrent falls |
| ROA | **0.70 (0.50 to 0.95)** | 5552 (1 study) | Moderate (imprecision) | ROA may have a very small effect for reducing risk of recurrent falls |
| *Fractures* |  |  |  |  |
| SOA | 0.93(0.66 to 1.30) | 418240 (4 studies) | Low (inconsistency, and publication bias) | SOA may have no effect for increasing risk of fractures |
| ROA | 1.05 (0.72 to 1.53) | 16516 (4 studies) | Low (inconsistency and publication) | ROA may have no effect for increasing risk of fractures |

*case-control and cross-sectional studies were removed.

CI: confidence interval; GRADE, Grading of Recommendations, Assessment, Development, and Evaluation. ROA: radiographic osteoarthritis; SOA: symptomatic osteoarthritis.

Inconsistency: downgraded because the proportion of variance in the effect estimates caused by true heterogeneity rather than chance is important (I^2^ >50%); Publication bias: funnel plot indicates a potential publication bias.

Table S4. Association of radiographic and symptomatic knee osteoarthritis with falls, recurrent falls and fractures. *

| **Outcomes** | **Odds ratio (95% CI)** | **№ of participants**  **(studies)** | **Certainty of the evidence**  **(GRADE)** | **Comments** |
| --- | --- | --- | --- | --- |
| *Falls* |  |  |  |  |
| SOA | 0.91 (0.44 to 1.87) | 734 (1 study) | Moderate (imprecision) | SOA may have no effect for increasing risk of falls |
| ROA | **1.39 (1.02 to 1.88)** | 734 (1 study) | Moderate (imprecision) | ROA may have a very small effect for increasing risk of falls |
| *Recurrent falls* |  |  |  |  |
| SOA | **1.55 (1.10 to 2.18)** | 2535 (1 study) | Moderate (imprecision) | SOA may have a small effect for increasing risk of recurrent falls |
| ROA | 1.39 (0.97 to 1.97) | 998 (2 studies) | Low (imprecision and publication bias) | ROA may have very small or no effect for increasing risk of recurrent falls |
| *Fractures* |  |  |  |  |
| SOA | **1.34 (1.15 to 1.57)** | 259112 (2 studies) | Low (risk of bias and publication bias) | SOA may have a small effect for increasing risk of fractures |
| ROA | 1.32 (0.74 to 2.34) | 1673 (2 studies) | Low (risk of bias and publication bias) | ROA may have no effect for increasing risk of fractures |

*studies that falls and fractures were self-reported were removing.

CI: confidence interval; GRADE, Grading of Recommendations, Assessment, Development, and Evaluation. ROA: radiographic osteoarthritis; SOA: symptomatic osteoarthritis.

Risk of bias: The case group definition for Cumming et.al [1] is based on self-report, with selection bias and no response rate not stated. Case group definition for Arden et.al [2] is based on self-report, with selection bias and control group selected from hospital. Inconsistency: downgraded because the proportion of variance in the effect estimates caused by true heterogeneity rather than chance is important (I^2^ >50%); Publication bias: funnel plot indicates a potential publication bias. Imprecision: only 1 study was available, and the wide CI may influence clinical decision.

Table S5. Associations of radiographic and symptomatic hip osteoarthritis with falls, recurrent falls and fractures. *

| **Outcomes** | **Odds ratio (95% CI)** | **№ of participants**  **(studies)** | **Certainty of the evidence**  **(GRADE)** | **Comments** |
| --- | --- | --- | --- | --- |
| *Falls* |  |  |  |  |
| SOA | 1.25 (0.95 to 1.65) | 8849 (3 studies) | Low (inconsistency and publication bias) | SOA may have very small or no effect for increasing risk of falls |
| Recurrent falls |  |  |  |  |
| *SOA* | **1.50 (1.28 to 1.75)** | 8087 (2 study) | Moderate (publication bias) | SOA may have a small effect for increasing risk of recurrent falls |
| ROA | **0.70 (0.50 to 0.95)** | 5552 (1 study) | Moderate (imprecision) | ROA may have a very small effect for reducing risk of recurrent falls |
| *Fractures* |  |  |  |  |
| SOA | 1.03 (0.56 to 1.88) | 264664 (3 studies) | Very low (risk of bias, inconsistency, and publication bias) | SOA may have no effect for increasing risk of fractures |
| ROA | 0.89 (0.27 to 2.93) | 9444 (3 studies) | Very low（risk of bias, inconsistency, and publication bias） | ROA may have no effect for reducing risk of fractures |

*studies that falls and fractures were self-reported were removing.

CI: confidence interval; GRADE, Grading of Recommendations, Assessment, Development, and Evaluation. ROA: radiographic osteoarthritis; SOA: symptomatic osteoarthritis.

Risk of bias: The case group definition for Cumming et.al [1] is based on self-report, with selection bias and no response rate not stated. Case group definition for Arden et.al [2] is based on self-report, with selection bias and control group selected from hospital. Inconsistency: downgraded because the proportion of variance in the effect estimates caused by true heterogeneity rather than chance is important (I^2^ >50%); Publication bias: funnel plot indicates a potential publication bias. Imprecision: only 1 study was available, and the wide CI may influence clinical decision.

| Outcomes | Odds ratio (95% CI) | № of participants  (studies) | Certainty of the evidence  (GRADE) | Comments |
| --- | --- | --- | --- | --- |
| *Fractures* |  |  |  |  |
| SHOA | 1.00 (0.70 to 1.51) | 5552 (1 study) | Moderate(imprecision) | SHOA may have no effect for risk of increasing fractures |
| RKOA | 0.72 (0.48 to 1.09) | 3005 (1 study) | Moderate(imprecision) | RKOA may have no effect for reducing risk of fractures |
| RHOA | 1.17 (0.87 to 1.59) | 8557 (2 studies) | Moderate (publication bias) | RHOA may have no effect for increasing risk of fractures |

Table S6. Evaluating the effect of radiographic and symptomatic hip and radiographic osteoarthritis on vertebral fractures.

CI: confidence interval; GRADE, Grading of Recommendations, Assessment, Development, and Evaluation, SKOA: symptomatic knee osteoarthritis, SHOA: symptomatic hip osteoarthritis, RKOA: radiographic osteoarthritis, RHOA: radiographic hip osteoarthritis.

Publication bias: funnel plot indicates a potential publication bias; Imprecision: only 1 study was available, and the wide CI may influence clinical decision.

# **Supplementary Figures**


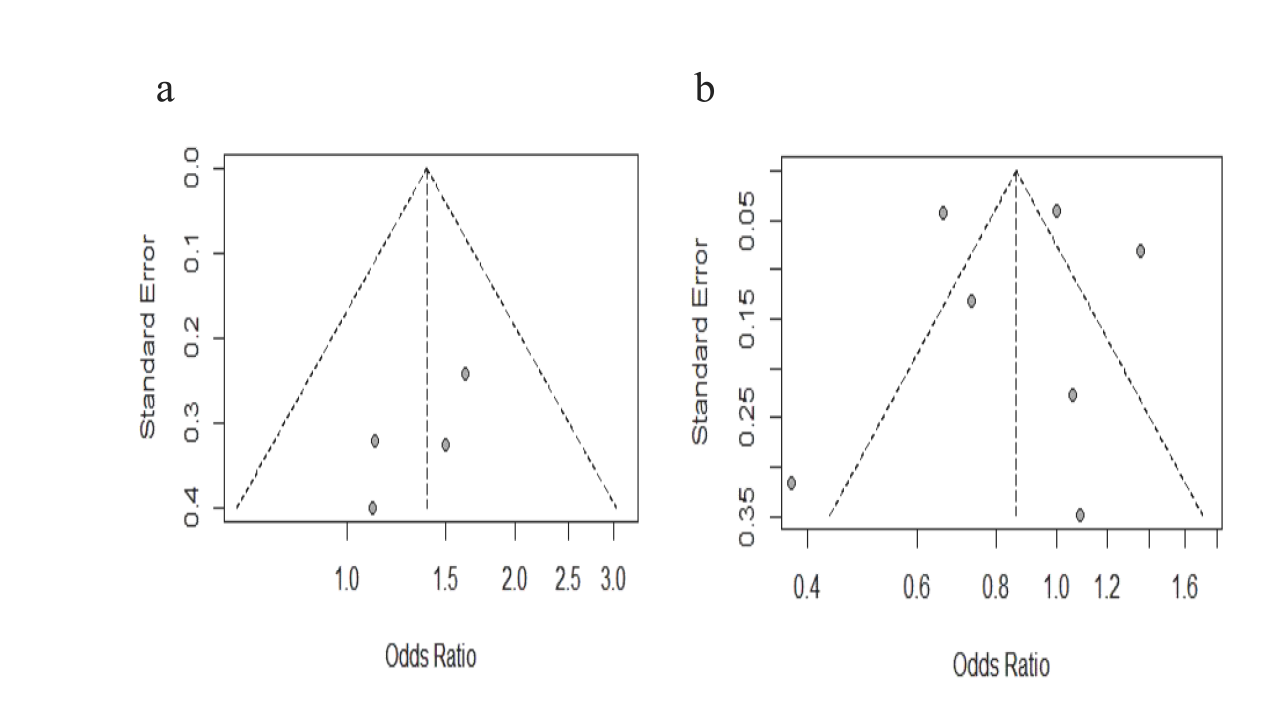


Figure S1. Funnel plots for the associations of radiographic and symptomatic knee osteoarthritis with falls and fractures. a: radiographic knee osteoarthritis and falls; b: symptomatic knee osteoarthritis and fractures.


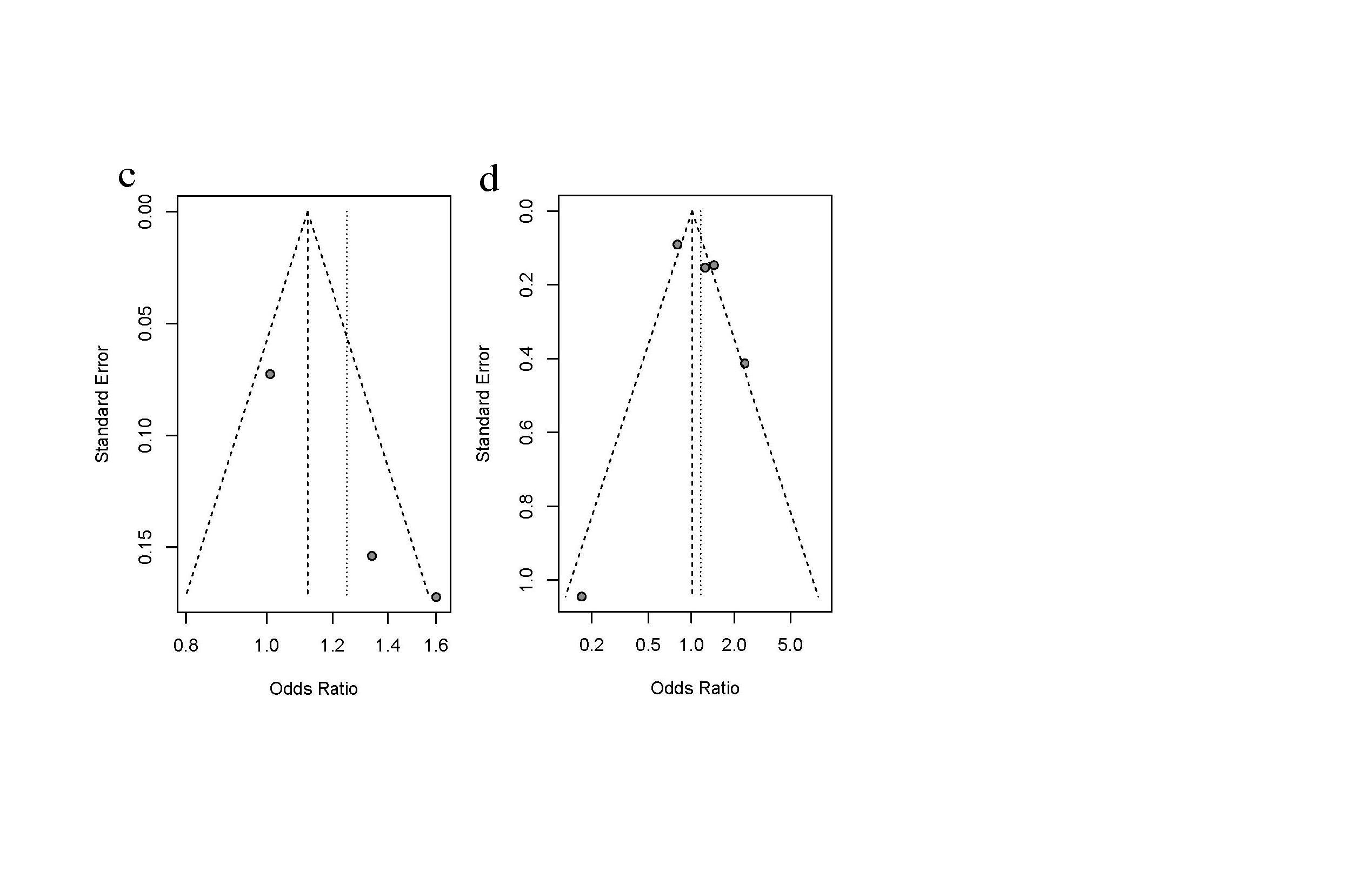


Figure S2. Funnel plots for the associations of radiographic and symptomatic hip osteoarthritis with falls and fractures. c: symptomatic knee osteoarthritis and falls; d: radiographic knee osteoarthritis and fractures.

References:

[1] Cumming RG, Klineberg RJ. Epidemiological study of the relation between arthritis of the hip and hip fractures. Ann Rheum Dis. 1993;52:707-10.

[2] Arden NK, Griffiths GO, Hart DJ, Doyle DV, Spector TD. The association between osteoarthritis and osteoporotic fracture: the Chingford Study. Br J Rheumatol. 1996;35:1299-304.
